# Supplementary material for: Impacts of ocean warming on fish size reductions on the world’s hottest coral reefs
Source: Nat Commun. 2024 Jul 1;15:5457. doi: 10.1038/s41467-024-49459-8 (PMC11217398; doi:10.1038/s41467-024-49459-8)
Supplement: Supplementary file 1 — Supplementary Information [file 41467_2024_49459_MOESM1_ESM.pdf]

Supplemental Information

Title:
Impacts of ocean warming on fish size reductions on the world's hottest coral reefs

Supplemental Figure *-Swimming respirometry:*

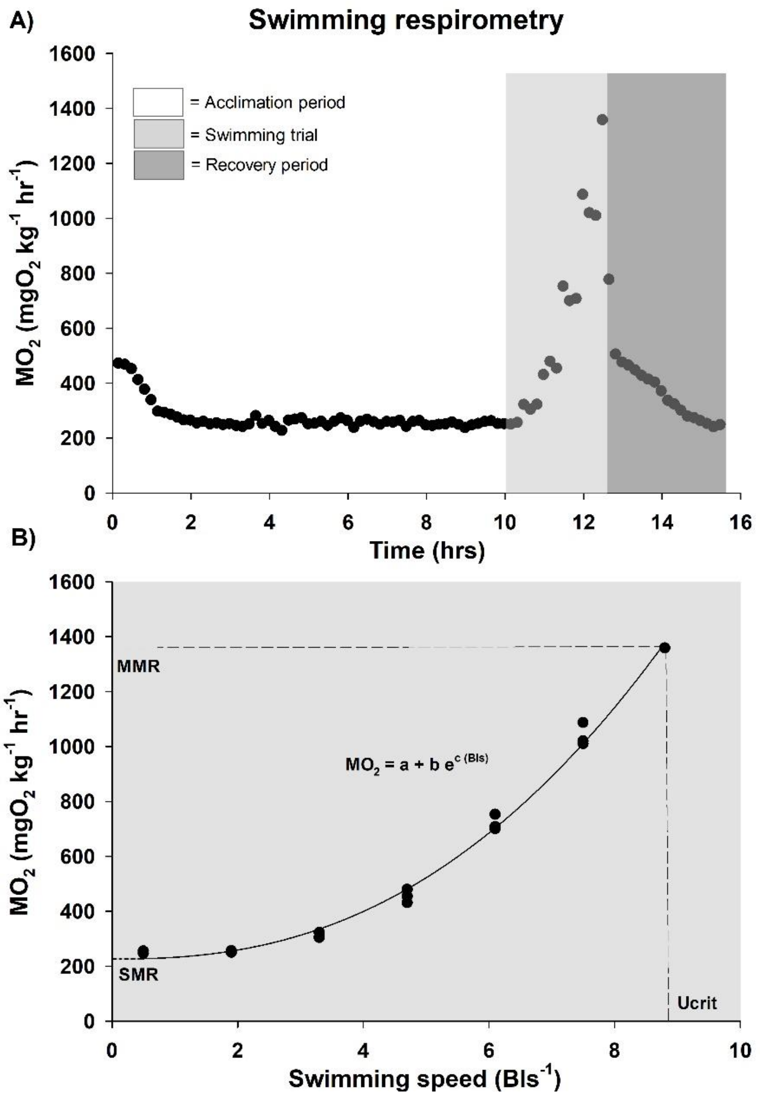

Supplemental Figure 1. Oxygen consumption (MO_2 in mgO_2 kg^{-1} hr^{-1}) of a fish inside a swimming respirometer during a 16hr trial period. Panel A shows MO_2 over time during the initial acclimation period (white section of graph), the subsequent swimming trial (light gray section) and final recovery period (dark gray section). Panel B shows MO_2 explicitly during the swimming trial from zero to maximal critical swimming speed (Ucrit) in body lengths per second (Bls^{-1}). Each black dot represents a single MO_2 value based on a 300s measuring period follow the intermittent flow respirometry methodology of Steffensen *et al.,* [94], Steffensen [95] and Svendsen *et al.,* [96]. Note, the horizontal stippled line in Panel B depicts maximum metabolic rate (MMR), which was taken as the highest recorded oxygen uptake across a single measuring period. The vertical stippled line in Panel B depicts Ucrit. Standard metabolic rate (i.e. SMR, oxygen uptake at rest) was calculated as the intercept with the y-axis at zero swimming speed using a nonlinear regression of oxygen uptake measures.

Supplemental Results

Detailed results for swimming energetics:

We predicted that species would show no improvements across regions. For *L. ehrenbergii*, the fixed effects explained 96% of the final model variance for metabolic performance. Overall, there was a significant interaction between temperature and all combined metabolic metrics examined for the two regions (i.e. AG versus GO SMR, MMR, AS, COT; $F_{6,111}$=2.245, p=0.044, Adj η^2p=0.060), but no direct effect of region ($F_{1,37}$=1.194, p=0.282, Adj η^2p=0.006). Post-hoc planned comparisons revealed that individuals from both regions showed significant increases in SMR, MMR, AS and COT with rising temperature between 27.0 and 35.5°C (see Figure 2). As predicted, these increases did not differ among regions at any temperature, and resulted in an average $Q10_{SMR}$=2.1 for both AG and GO individuals (Figure 2).

For *S. ghanam*, the fixed effects explained 89% of the final model variance. Overall, there was a significant interaction between temperature and all combined metabolic metrics examined for the two regions (i.e. SMR, MMR, AS, COT; $F_{3,105}$=10.324, p<0.001, Adj η^2p=0.210), and a significant regional effect ($F_{1,35}$=6.307, p=0.017, Adj η^2p=0.130). Post-hoc planned comparisons revealed that there were no significant differences in SMR or COT among regions from 27.0 - 31.5°C (Figure 2), and neither region showed a significant increase in SMR with rising temperature, resulting in a mean $Q10_{SMR}$=1.45 (Figure 2). The two regions did, however, differ in both MMR and AS at 31.5°C (t_{MMR}=5.316, p<0.001; t_{AS}=6.215, p<0.001) and 35.5°C. Specifically, AG individuals showed a significant peak in MMR and AS at 31.5°C (27.0-31.5°C: t_{MMR}=-3.275, p=0.001, t_{AS}=3.083, p=0.003; 31.5-35.5°C: t_{MMR}=3.777, p<0.001, t_{AS}=4.743, p<0.001) and no significant differences in these metrics between 27.0°C and 35.5°C (t_{MMR}=0.739, p=0.462; t_{AS}=1.855, p=0.066). Contrary to predictions, GO individuals differed from AG individuals by showing no significant change in MMR from 27.0°C to 31.5°C (t=1.531, p=0.129), a significant reduction in AS across the same temperatures (t=2.847, p=0.005), and none of these fishes could be acclimated to the 35.5°C treatment.

Detailed results for swimming performance:

We predicted that species would show no improvements across regions. For *L. ehrenbergii*, the fixed effects explained 65% of the final model variance for swimming performance. Overall, there was no significant interaction between temperature and all combined swimming metrics examined for the two regions (i.e. U_{crit}, U_{burst}, U_{opt}; $F_{4,74}$=1.308, p=0.275, Adj η^2p=0.020), and no direct regional effect ($F_{1,37}$=0.373, p=0.546, Adj η^2p=0.000). Post-hoc planned comparisons also revealed no significant changes in U_{burst}, U_{crit} or U_{opt} neither within or across temperatures or between regions (see Figure 3).

For *S. ghanam*, the fixed effects explained 67% of the final model variance. There was a significant effect of region ($F_{1,35}$=19.380, p<0.001, Adj η^2p=0.340) and interaction between temperature and swimming metrics ($F_{4,68}$=15.170, p<0.001, Adj η^2p=0.440). Contrary to predictions, post-hoc planned comparisons showed that the two regions differed significantly in swimming performance: The AG individuals had significantly higher U_{crit}, U_{burst} and U_{opt} at 31.5°C (t=5.492, p<0.001; t=4.492, p<0.001; t=3.017, p=0.004, respectively. Figure 3). The AG individuals also showed no change in swimming performance from 27.0-31.5°C and was able to swim consistently at 35.5°C where no GO individual could be tested (Figure 3). In comparison, the GO individuals suffered significant reductions in U_{crit}, U_{burst} and U_{opt} with rising temperature from 27.0 - 31.5°C ranging from 16.6 – 22.4% (t=3.511, p=0.001; t=2.717, p=0.009; t=2.289, p=0.027, respectively. Figure 3).

Detailed results for kinematic muscle performance:

We predicted that species would show no improvements across regions. For *L. ehrenbergii* there was a significant interaction between temperature, region and swimming speed for Amp ($F_{2,99}$=3.862, p=0.024, Adj η^2p=0.050) and St ($F_{2,95}$=4.818, p=0.010, Adj η^2p=0.07), and between temperature and swimming speed for Freq ($F_{2,96}$=5.243, p=0.007, Adj η^2p=0.080). Within temperature comparisons of responses across swimming speeds showed no significant difference among regions for any temperature (Amp: $t_{27.0°C}$=-2.127, p=0.179, $t_{31.5°C}$=1.320, p=0.356, $t_{35.5°C}$=-1.491, p=0.348; Freq: $t_{27.0°C}$=-0.512, p=0.863, $t_{31.5°C}$=0.247, p=0.863, $t_{35.5°C}$=-0.776, p=0.733; St: $t_{27.0°C}$=-1.733, p=0.246, $t_{31.5°C}$=2.865, p=0.051, $t_{35.5°C}$=0.159, p=0.960). At all temperatures, pooled AG and GO data revealed an increase in Amp and Freq with increasing swimming speeds (Amp: $t_{27.0°C}$=4.564, p<0.001, $t_{31.5°C}$=7.326, p<0.001, $t_{35.5°C}$=4.356, p<0.001; Freq: $t_{27.0°C}$=11.168, p<0.001, $t_{31.5°C}$=16.725, p<0.001, $t_{35.5°C}$=18.324, p<0.001), and a reduction in St with increasing swimming speeds ($t_{27.0°C}$=-4.290, p<0.001, $t_{31.5°C}$=-3.993, p<0.001, $t_{35.5°C}$=-3.514, p<0.001). There were no changes in slopes across temperatures for Amp or St (see Fig. 4), and a steeper slope of Hz at 35.5°C than 27.0°C and 31.5°C ($t_{27.0-31.5°C}$=-0.026, p=0.979, $t_{27.0-35.5°C}$=-2.424, p=0.026, $t_{31.5-35.5°C}$=-2.962, p=0.012).

For *S. ghanam* there was a significant interaction between region and swimming speed for Amp ($F_{1,116}$=14.468, p<0.001, Adj η^2p=0.100) and a direct effect of swimming speed on Freq ($F_{1,121}$=39.756, p<0.001, Adj η^2p=0.240) and St ($F_{1,118}$=71.071, p<0.001, Adj η^2p=0.37). Within temperature comparisons showed a significant difference in the slope of Amp across swimming speeds between AG and GO at 27.0°C (t=-4.574, p<0.001) but not 31.5°C (t=-1.686, p=0.203). At all temperatures there was a significant increase in Amp with increasing swimming speed (AG: $t_{27.0°C}$=5.301, p=0<0.001, $t_{31.5°C}$=9.004, p<0.001, $t_{35.5°C}$=3.273, p=0.001; GO: $t_{27.0°C}$=9.934, p<0.001, $t_{31.5°C}$=5.238, p<0.001). Within temperature comparisons of Freq and St across swimming speeds showed no significant difference among regions for any temperature (Freq: $t_{27.0°C}$=1.556, p=0.122, $t_{31.5°C}$=0.275, p=0.784; St: $t_{27.0°C}$=-0.070, p=1.000, $t_{31.5°C}$=0.955, p=0.853). At all temperatures, pooled AG and GO data revealed an increase in Freq with increasing swimming speeds (Freq: $t_{27.0°C}$=6.446, p<0.001, $t_{31.5°C}$=6.481, p<0.001, $t_{35.5°C}$=3.424, p<0.001), and a reduction in St with increasing swimming speeds ($t_{27.0°C}$=-8.336, p<0.001, $t_{31.5°C}$=-5.404, p<0.001, $t_{35.5°C}$=-4.722, p<0.001). There were no changes in slopes across temperatures for Freq or St (see Fig. 4).

Detailed results for effect of temperature on mass-scaling:

We predicted that larger fish should experience greater reductions in performance with each degree increase in temperature. For *L. ehrenbergii* there was a significant effect of mass on AS ($F_{1,31}$=31.807, p<0.001, Adj η^2p=0.490), but no direct or interactive effect with temperature or region (three-way interaction: $F_{2,31}$=1.053, p=0.361, Adj η^2p=0.003). A within temperature comparison of mass-scaling slopes showed no significant difference among regions for any temperature ($t_{27.0°C}$=1.456, p=0.762, $t_{31.5°C}$=1.115, p=0.762, $t_{35.5°C}$=-0.410, p=0.929). Pooled AG and GO data revealed a positive increase in AS with increasing mass at all temperatures ($t_{27.0°C}$=11.001, p<0.001, $t_{31.5°C}$=6.132, p<0.001, $t_{35.5°C}$=10.922, p<0.001), and no change in mass-scaling slopes across temperatures ($t_{27.0-31.5°C}$=0.671, p=0.508, $t_{27.0-35.5°C}$=-0.872, p=0.508, $t_{31.5-35.5°C}$=-1.321, p=0.508, see Figure 5).

For U_{crit}, there was no significant effect of mass ($F_{1,31}$=0.014, p=0.906, Adj η^2p=0.000) and no direct or interaction effect of mass with temperature or region (three-way interaction: $F_{2,31}$=0.224, p=0.801, Adj η^2p=0.000). A within temperature comparison of mass-scaling slopes showed no significant difference among regions for any temperature ($t_{27.0°C}$=-0.222, p=0.884, $t_{31.5°C}$=1.250, p=0.539, $t_{35.5°C}$=0.687, p=0.710). Pooled AG and GO data showed a significant increase in U_{crit} with increasing mass at 27.0°C, but not an any other temperature ($t_{27.0°C}$=-3.627, p<0.001, $t_{31.5°C}$=-0.972, p=0.338, $t_{35.5°C}$=0.099, p=0.926)

and no change in mass-scaling slopes across temperatures ($t_{27.0\text{-}31.5°C}$=-0.697, p=0.561, $t_{27.0\text{-}35.5°C}$=-1.259, p=0.561, $t_{31.5\text{-}35.5°C}$=-0.600, p=0.561, Figure 5).

For SMR there was a significant interaction between mass and temperature ($F_{2,31}$=5.595, p=0.008, Adj η^2p=0.220), but no direct or interaction effect of mass with temperature or region (three-way interaction: $F_{2,31}$=0.654, p=0.527, Adj η^2p=0.000). A within temperature comparison of mass-scaling slopes showed no significant difference among regions for any temperature ($t_{27.0°C}$=1.052, p=0.441, $t_{31.5°C}$=-0.126, p=0.951, $t_{35.5°C}$=0.712, p=0.602). Pooled data showed a positive increase in SMR with mass at all temperatures ($t_{27.0°C}$=5.011, p<0.001, $t_{31.5°C}$=3.668, p<0.001, $t_{35.5°C}$=4.602, p<0.001), and no change in mass-scaling slopes across temperatures ($t_{27.0\text{-}31.5°C}$=-0.126, p=0.900, $t_{27.0\text{-}35.5°C}$=-2.417, p=0.055, $t_{31.5\text{-}35.5°C}$=-2.185, p=0.055, see Figure 5).

Similarly, COT showed a significant interaction between mass and temperatures ($F_{2,31}$=18.962, p<0.001, Adj η^2p=0.520), but no direct or interactive effect of mass with temperature or region (three-way interaction: $F_{2,31}$=2.388, p=0.109, Adj η^2p=0.080). A within temperature comparison of mass-scaling slopes showed no significant difference among regions for any temperature ($t_{27.0°C}$=1.308, p=0.273, $t_{31.5°C}$=-1.064, p=0.341, $t_{35.5°C}$=-1.949, p=0.101). Pooled data showed a positive increase in COT with mass at all temperatures ($t_{27.0°C}$=6.308, p<0.001, $t_{31.5°C}$=5.319, p<0.001, $t_{35.5°C}$=7.125, p<0.001), and a steeper slope at 35.0°C than at 27.0°C and 31.5°C ($t_{27.0\text{-}31.5°C}$=-0.733, p=0.469, $t_{27.0\text{-}35.5°C}$=-4.310, p<0.001, $t_{31.5\text{-}35.5°C}$=-3.599, p=0.002, see Figure 6), highlighting that contrary to predictions, *L. ehrenbergii* was not limited by mass at elevated temperatures and there were no recorded negative consequences for larger individuals.

For *S. ghanam* there was a significant interaction between temperature and mass on AS ($F_{1,29}$=20.873, p<0.001, Adj η^2p=0.400), but no direct or interactive effect of mass with temperature or region (three-way interaction: $F_{1,29}$=0.075, p=0.787, Adj η^2p=0.000). A within temperature comparison of mass-scaling slopes showed no significant difference among regions at either temperature ($t_{27.0°C}$=0.380, p=1.00, $t_{31.5°C}$=0.090 p=1.000). Pooled AG and GO data revealed a significant increase in AS with increasing mass at 27.0°C and 31.5°C ($t_{27.0°C}$=10.196, p<0.001, $t_{31.5°C}$=3.005, p=0.005), and a significant reduction in slopes from 27.0°C to 31.5°C (t=2.715, p=0.016). AG data at 35.5°C showed no significant effect of mass on AS ($t_{35.5°C}$=0.365, p=0.718) and a reduction in slope from 27.0°C ($t_{27.0\text{-}35.5°C}$=2.961 p=0.016, $t_{31.5\text{-}35.0°C}$=1.162, p=0.719, see Figure 5).

For U_{crit}, there was a significant interaction between mass and temperature ($F_{2,29}$=4.892, p=0.015, Adj η^2p=0.200), but no interaction region (three-way interaction: $F_{1,29}$=1.135, p=0.296, Adj η^2p=0.004). A within temperature comparison of mass-scaling slopes showed no significant difference among regions for any temperature ($t_{27.0°C}$=-0.293, p=1.000, $t_{31.5°C}$=-2.468, p=0.0.099). Pooled AG and GO data showed no effect of mass at 27.0°C ($t_{27.0°C}$ =1.145, p=0.261) but a significant reduction in U_{crit} with increasing mass at 31.5°C (t=-2.953, p=0.006). Pooled data confirmed a reduction in mass-scaling slopes from 27.0°C to 31.5°C (t=3.120, p=0.011). AG data at 35.5°C showed no significant effect of mass on U_{crit} ($t_{35.5°C}$=-0.227, p=0.822) and no difference in slope from other treatment temperatures ($t_{27.0\text{-}35.5°C}$=0.587, p=0.561, $t_{31.5\text{-}35.5°C}$=-1.261, p=0.324, Figure 5).

For SMR, there was a significant effect of mass ($F_{1,30}$=32.164, p<0.001, Adj η^2p=0.500), but no direct or interactive effect of mass with temperature or region (three-way interaction: $F_{1,30}$=0.367, p=0.549, Adj η^2p=0.000). A within temperature comparison of mass-scaling slopes showed no significant difference among regions for any temperature ($t_{27.0°C}$=-0.861, p=0.990, $t_{31.5°C}$=-0.241, p=1.000). After pooling 27.0 and 31.5°C data, there was a significant positive increase in SMR with mass at all temperatures ($t_{27.0°C}$=7.073, p<0.001, $t_{31.5°C}$=5.677, p<0.001, $t_{35.5°C}$=4.267, p<0.001) and no difference in mass-scaling

slopes across temperatures ($t_{27.0\text{-}31.5°C}$=-0.782, p=0.444, $t_{27.0\text{-}35.5°C}$=-1.627, p=0.340, $t_{31.5\text{-}35.5°C}$=-1.079, p=0.432, Figure 5).

Similarly, COT showed an effect of mass ($F_{1,30}$=37.425, p<0.001, Adj η^2p=0.540) but no direct or interactive effect of region (three-way interaction: $F_{1,30}$=0.011, p=0.916, Adj η^2p=0.000). A within temperature comparison of mass-scaling slopes showed no significant difference among regions for any temperature ($t_{27.0°C}$=0.183, p=1.000, $t_{31.5°C}$=0.497, p=1.000). After pooling, data showed a significant positive increase in COT with mass at all temperatures ($t_{27.0°C}$=-6.957, p<0.001, $t_{31.5°C}$=-4.594, p<0.001, $t_{35.5°C}$=-3.001, p=0.005), and no significant difference between temperatures ($t_{27.0\text{-}31.5°C}$=0.474, p=0.641, $t_{27.0\text{-}35.5°C}$=1.089, p=0.624, $t_{31.5\text{-}35.5°C}$=0.823, p=0.624, Figure 6), highlighting that *S. ghanam* was partially limited by mass at elevated temperatures within a subset of performance metrics.
