# Supplementary material for: Impacts of ocean warming on fish size reductions on the world’s hottest coral reefs
Source: Nat Commun. 2024 Jul 1;15:5457. doi: 10.1038/s41467-024-49459-8 (PMC11217398; doi:10.1038/s41467-024-49459-8)
Supplement: Supplementary file 3 — Description of Additional Supplementary Files [file 41467_2024_49459_MOESM3_ESM.pdf]

Description of Additional Supplementary Files

This section contain legends for each Supplementary Data

Supplementary Data 1.
Source Data and Code

Supplementary Data 2.
Parameter estimates and test statistics from models of *Lutjanus ehrenbergii* 1) metabolic metrics (standard metabolic rate [SMR], maximum metabolic rate [MMR], absolute aerobic scope [AS], cost-of-transport [COT]); 2) swimming metrics (burst speed [Uburst], optimal speed [Uopt], critical speed [Ucrit]); 3) kinematic metrics (fin beat amplitude [Amp], frequency [Freq], Strouhal number [St]); and 4) mass scaling of SMR, AS, COT, and Ucrit. Model structures presented are those supported by model selection. A total of 44 *L. ehrenbergii* were used for final analyses, equating to 11, 16, and 16 individuals at 27.0, 31.5 and 35.5°C respectively. Note, False Detection Rate corrections were used to account for Type I error among planned contrasts and significant terms are indicated in bold.

Supplementary Data 3.
Parameter estimates and test statistics from models of *Scolopsis ghanam* 1) metabolic metrics (standard metabolic rate [SMR], maximum metabolic rate [MMR], absolute aerobic scope [AS], cost-of-transport [COT]); 2) swimming metrics (burst speed [Uburst], optimal speed [Uopt], critical speed [Ucrit]); 3) kinematic metrics (fin beat amplitude [Amp], frequency [Freq], Strouhal number [St]); and 4) mass scaling of SMR, AS, COT, and Ucrit. Model structures presented are those supported by model selection. A total of 40 *S. ghanam* were used for final analyses, equating to 17, 17, and 6 individuals at 27.0, 31.5 and 35.5°C respectively. Note, False Detection Rate corrections were used to account for Type I error among planned contrasts and significant terms are indicated in bold.

Supplementary Data 4.
Comparison of body size within treatments for *Lutjanus ehrenbergii* and *Scolopsis ghanam* from the Persian / Arabian Gulf (AG) and Gulf of Oman (GO). Temperature is in degree Celsius (°C), mass is in gram and standard length (SL) is in cm. Significant differences are highlighted in bold.
