# Supplementary material for: Impacts of ocean warming on fish size reductions on the world’s hottest coral reefs
Source: Nat Commun. 2024 Jul 1;15:5457. doi: 10.1038/s41467-024-49459-8 (PMC11217398; doi:10.1038/s41467-024-49459-8)
Supplement: Supplementary file 8 — Reporting Summary [file 41467_2024_49459_MOESM8_ESM.pdf]

nature portfolio

Corresponding author(s): Jacob Johansen

Last updated by author(s): Apr 8, 2024

Reporting Summary

Nature Portfolio wishes to improve the reproducibility of the work that we publish. This form provides structure for consistency and transparency in reporting. For further information on Nature Portfolio policies, see our Editorial Policies and the Editorial Policy Checklist.

Statistics

For all statistical analyses, confirm that the following items are present in the figure legend, table legend, main text, or Methods section.

n/a	Confirmed	
☐	☒	The exact sample size (*n*) for each experimental group/condition, given as a discrete number and unit of measurement
☐	☒	A statement on whether measurements were taken from distinct samples or whether the same sample was measured repeatedly
☐	☒	The statistical test(s) used AND whether they are one- or two-sided<br>*Only common tests should be described solely by name; describe more complex techniques in the Methods section.*
☐	☒	A description of all covariates tested
☐	☒	A description of any assumptions or corrections, such as tests of normality and adjustment for multiple comparisons
☐	☒	A full description of the statistical parameters including central tendency (e.g. means) or other basic estimates (e.g. regression coefficient) AND variation (e.g. standard deviation) or associated estimates of uncertainty (e.g. confidence intervals)
☐	☒	For null hypothesis testing, the test statistic (e.g. *F*, *t*, *r*) with confidence intervals, effect sizes, degrees of freedom and *P* value noted<br>*Give P values as exact values whenever suitable.*
☒	☐	For Bayesian analysis, information on the choice of priors and Markov chain Monte Carlo settings
☐	☒	For hierarchical and complex designs, identification of the appropriate level for tests and full reporting of outcomes
☐	☒	Estimates of effect sizes (e.g. Cohen's *d*, Pearson's *r*), indicating how they were calculated

Our web collection on statistics for biologists contains articles on many of the points above.

Software and code

Policy information about availability of computer code

Data collection	Oxygen consumption were measured using fibre optic oxygen meters and monitored with AutoResp V2 (Loligo Systems, Denmark) and AquaResp V3 (Pyroscience Firesting sensors, Germany). Kinematic data were collected using LoggerPro 3.14.1 (Vernier, USA)
Data analysis	The statistical analyses included in this manuscript were all conducted in R-studio 2022.12.0 using R-version 4.1.2 or above on Windows 11 (64 bit) machines. All data were analyzed using the packages ‘lme4’, ‘lmerTest’, ‘MuMIn’, ‘multcomp’, ‘languageR’, ‘LMERConvenienceFunctions’, ‘emmeans’, ‘EnvStats’, ‘outliers’, ‘effectsize’, r2glmm’ and ‘car’. All softwares and codes are open source and provided as a supplemental zip file with this publication. Graphs were completed using SigmaPlot V.14.

For manuscripts utilizing custom algorithms or software that are central to the research but not yet described in published literature, software must be made available to editors and reviewers. We strongly encourage code deposition in a community repository (e.g. GitHub). See the Nature Portfolio guidelines for submitting code & software for further information.

Data

Policy information about availability of data

All manuscripts must include a data availability statement. This statement should provide the following information, where applicable:

- Accession codes, unique identifiers, or web links for publicly available datasets
- A description of any restrictions on data availability
- For clinical datasets or third party data, please ensure that the statement adheres to our policy

All source data and analytical code are provided as a supplemental file with this publication

Research involving human participants, their data, or biological material

Policy information about studies with human participants or human data. See also policy information about sex, gender (identity/presentation), and sexual orientation and race, ethnicity and racism.

Reporting on sex and gender	N/A
Reporting on race, ethnicity, or other socially relevant groupings	N/A
Population characteristics	N/A
Recruitment	N/A
Ethics oversight	N/A

Note that full information on the approval of the study protocol must also be provided in the manuscript.

Field-specific reporting

Please select the one below that is the best fit for your research. If you are not sure, read the appropriate sections before making your selection.

☐ Life sciences ☐ Behavioural & social sciences ☒ Ecological, evolutionary & environmental sciences

For a reference copy of the document with all sections, see nature.com/documents/nr-reporting-summary-flat.pdf

Ecological, evolutionary & environmental sciences study design

All studies must disclose on these points even when the disclosure is negative.

Study description	A comparison of 10 performance metrics for two species, each across two regions and three temperatures. A total of 83 fishes were used for final analyses, providing 5-10 independent replicates per treatment. Analyses were conducted using three statistical approaches: 1) Energetics: fully factorial linear mixed effect models with temperature, region and performance metric (i.e. standard metabolic rate, maximum metabolic rate, aerobic scope, cost-of-transport, critical swimming speed, burst-coast swimming speed, optimal swimming speed) as the fixed factors and individual ID as a random effect; 2) Kinematics: fully factorial linear mixed effect models with temperature, region and performance metric (i.e. amplitude, frequency, strouhal number) as fixed effects, individual ID as a random effect, and swimming speed as a continuous covariate; 3) Size effect: fully factorial linear mixed effect model with least squares trend for estimating and comparing mass-scaling slopes within and across temperatures and regions. Each model included temperature and region as random nested effects, and mass as a continuous covariate to account for sampling variance. Where region trends did not differ within temperatures, data were pooled to increase statistical power, followed by post-hoc planned comparisons.
Research sample	Reef fishes from two families were selected as good indicators of thermal adaptation potential in reef fishes. The species Lutjanus ehrenbergii and Scolopsis ghanam were chosen for study since they are among the most abundant fishes in the AG and GO study regions while also representing evolutionary distinct lineages (Lutjanidae and Nemipteridae). In addition, the chosen species are piscivorous / omnivorous and are by way of feeding mode forced to swim when foraging. By comparing individuals from three AG and three GO sites representing the divergent thermal AG and GO environments, these fishes provided the opportunity to evaluate consequences of elevated temperatures on a range of performance metrics within closely related populations (intraspecies) as well as across evolutionary traits (interspecies).
Sampling strategy	A total of 85 fishes (Lutjanus: n = 44, 12.3 ± 0.3 cm standard length (SL), range 9.2 – 16.5 cm SL, 48.1 ± 3.36 g, range 20.0 – 116.1 g; Scolopsis: n = 41, 11.5 ± 0.2 cm SL, range 9.2 – 14.2 cm, 40.2 ± 2.4 g, range 16.6 – 87.4 g; mean ± S.E.M.) were collected by scuba divers using fine-mesh monofilament barrier nets. Only adult fish were used in this study to avoid ontogenetic differences, and collected size ranges were matched across AG and GO to the greatest extent possible. All collections were conducted during periods of comparable ambient water temperatures (i.e. when SST was 27.0 ± 0.5°C, 31.5 ± 0.5°C, 35.5 ± 0.5°C in the AG region; and 27.0 ± 0.5°C, 31.5 ± 0.5°C in the GO region). These temperatures corresponded to the approximate annual mean and summer max

	temperatures found in AG (27.0 and 35.5°C) and GO (27.0 and 31.5°C) regions. Cardio-respiratory studies date back over 60 years and in fishes consistently require 5-10 individuals per treatment for statistical strength, and those numbers were therefore collected for each species.
Data collection	Data collection was conducted by two postdoctoral researchers and one Ph.D. student. Oxygen consumption were measured using fibre optic oxygen meters and monitored with AutoResp V2 (Loligo Systems, Denmark) and AquaResp V3 (Pyroscience Firesting sensors, Germany). Kinematic data were collected using LoggerPro 3.14.1 (Vernier, USA).<br>Specifically, At each temperature the oxygen uptake and swimming ability (mode, speed and kinematics) of each species was quantified for individuals swimming solitarily in a 10L clear Plexiglas swim-tunnel respirometer. Two identical Steffensen-type respirometers were used simultaneously, each with a working section of 8.0 x 8.0 x 10.0 cm (length x width x depth). Individuals were assigned to each respirometer at random and were unable to see one another. Flow within the working section of the respirometers was calibrated from 0 to 125.0 ± 0.5 cm s-1 (mean ± SE) using a digital TAD W30 flow-meter (Hoentzsch, Germany). Solid blocking effects of the fish in the working section were corrected following Bell & Terhune 90 and were kept below 5%.<br>At the beginning of each trial, a respirometer was filled with temperature controlled (27.0, 31.5 or 35.5 ± 0.1ºC, mean ± SE), filtered and fully aerated seawater. Next, a fish was placed in the respirometer and left to acclimatize for ~8 h at a swimming speed of ~0.5 body length per second average (bl s-1), until oxygen uptake of the test subject reached a steady state level and the fish had settled into a continuous slow swimming rhythm. The trial was then started and the oxygen uptake of the test subject was measured at increasing swimming speeds for a total of 30 min at every speed, using 0.6-1.4 bls-1 speed increments. The maximum swimming speed tested was dependent on the swimming ability of the individual fish, with flow velocities incrementally increasing until the fish could no longer keep up and was swept downstream with the flow onto a retaining grid for longer than 5 s. At this point the flow velocity and total swimming time was recorded, and the fish returned to a 0.5 bls-1 swimming speed to recover. Once oxygen uptake reduced to a steady state level (within 20% of the oxygen uptake at beginning of the experiment), the fish was deemed to have recovered, the experiment was stopped and the fish was returned to its holding tank. During the trial, the fish was continuously monitored for swimming mode (i.e., steady versus burst swimming), and the total swimming time and flow speed was recorded at the point of change from steady caudal swimming to burst-and-coast propulsion for longer than 5 s continuously.<br>For every oxygen measurement, a dynamic ~240 s flush, ~60 s equilibration and ~300 s measurement period was applied. The flushing period ensured the oxygen concentration throughout the trial did not decrease below 80% of air saturation and reduced any CO2 build up.
Timing and spatial scale	From January 2018 to July 2020. All collections were conducted during periods of comparable ambient water temperatures (i.e. when SST was 27.0 ± 0.5°C, 31.5 ± 0.5°C, 35.5 ± 0.5°C in the AG region; and 27.0 ± 0.5°C, 31.5 ± 0.5°C in the GO region). These temperatures corresponded to the approximate annual mean and summer max temperatures found in AG (27.0 and 35.5°C) and GO (27.0 and 31.5°C) regions, which are separated by approximately 450km. Exact collection locations: Dhabiya (24.36383º, 54.10121º), Ras Ghanada (24.84743º, 54.69235º), Saadiyat (24.65771º, 54.48691º), Dibba Rock (25.55378º, 56.35694º), Sharm Rock (25.48229º, 56.36695º), Snoopy Rock (25.49210º, 56.36401º).
Data exclusions	All model data were tested for univariate assumptions using Shapiro-Wilk normality test, Levene's test for homogeneity of variance and Grubb's outlier test, which define outliers based on the largest absolute deviation from the sample mean. Data that did not initially comply with assumptions were Box-Cox transformed and a total of two single data points were removed as outliers (out of >1800 data points). As L.enhrenbergii displayed unsteady swimming at speeds ≤1.9 bls and S. ghanam at ≤1.1 bls, species-specific kinematics could not be accurate evaluated at these speeds and were excluded from kinematic analyses. All final model data met assumptions.
Reproducibility	The experimental findings presented here are generalized patterns from two independent species by exposing 5-10 independent individuals of each species from each region to each of the three temperature treatments. The experiments were performed on a total of 85 individuals.
Randomization	All individuals in this study underwent the same single trial, during which 10 performance metrics were examined simultaneously.
Blinding	Specimen trial dates were randomized and all data collection were conducted using automatic softwares and standardized protocols (thus preventing potential bias). Only kinematic variables were manually extracted using LoggerPro, for which the experimenter was kept blind to the origin region of the fish whenever possible.

Did the study involve field work? ☒ Yes ☐ No

Field work, collection and transport

Field conditions	Shallow water coral reef ecosystems. Water temperature 27-36C. Air temperature 25-50C.
Location	Six reef sites in the southeastern region of the Arabian Gulf (AG) and northwestern region of the Gulf of Oman (GO). Reef sites AG: Dhabiya: 24.36383º, 54.10121º; Ras Ghanada: 24.84743º, 54.69235º; Saadiyat: 24.65771º, 54.48691º. Reef sites GO: Dibba Rock: 25.55378º, 56.35694º; Sharm Rock: 25.48229º, 56.36695º; Snoopy Rock: 25.49210º, 56.36401º.
Access & import/export	All collections and trials were conducted under animal ethics permit IACUC 17-0002, 20-0001 and Environment Abu Dhabi, UAE collection permit TMBS/17/l/284.
Disturbance	Collections were explicit to the study species (i.e. no other species were impacted) and numbers were kept to the minimum required for statistical validity (as required by permitting agencies).

Reporting for specific materials, systems and methods

We require information from authors about some types of materials, experimental systems and methods used in many studies. Here, indicate whether each material, system or method listed is relevant to your study. If you are not sure if a list item applies to your research, read the appropriate section before selecting a response.

Materials & experimental systems

n/a	Involved in the study
☒	☐ Antibodies
☒	☐ Eukaryotic cell lines
☒	☐ Palaeontology and archaeology
☐	☒ Animals and other organisms
☒	☐ Clinical data
☒	☐ Dual use research of concern
☒	☐ Plants

Methods

n/a	Involved in the study
☒	☐ ChIP-seq
☒	☐ Flow cytometry
☒	☐ MRI-based neuroimaging

Animals and other research organisms

Policy information about studies involving animals; ARRIVE guidelines recommended for reporting animal research, and Sex and Gender in Research

Laboratory animals	No laboratory animals were used in this study
Wild animals	Reef fishes from two families were selected as good indicators of thermal adaptation potential in reef fishes. The species Lutjanus ehrenbergii and Scolopsis ghanam were chosen for study since they are among the most abundant fishes in AG and GO while also representing evolutionary distinct lineages (Lutjanidae and Nemipteridae). Fish were collected by scuba divers using fine-mesh monofilament barrier nets. Only adult fish were used in this study to avoid ontogenetic differences, and collected size ranges were matched across AG and GO to the greatest extent possible. After collection, fishes were transported by boat and car to the seawater laboratory facilities at New York University Abu Dhabi (NYUAD). After trials were completed, all fish were kept for additional studies (unrelated to this publication).
Reporting on sex	This manuscript does not include cell-lines of fish and these species cannot be sexually differentiated without euthanasia and dissection. Our study examines broad-universal responses to temperature that are unrelated to sex
Field-collected samples	All fish were held in 80 x 40 x 40 cm tanks (length x width x height) under a 12 – 12 h light-dark regime (subjected to sunrise as beginning of daylight) in groups of four fish per tank to allow individual identification. Tanks were continuously supplied with filtered seawater from individual sumps (containing a protein skimmer and canister filter to maintain water quality), at the ambient collection temperature of 27.0, 31.5, or 35.5ºC (mean ± 0.1ºC) and 40ppt salinity, equivalent to the mean ppt in AG and GO sites. All fish were fed twice daily to satiation with commercial fish foods and left undisturbed to settle to lab conditions until regular feeding patterns were observed (7-10 days). After trials were completed, all fish were kept for additional studies (unrelated to this publication).
Ethics oversight	New York University Abu Dhabi institutional animal care and use committee (IACUC)

Note that full information on the approval of the study protocol must also be provided in the manuscript.

Plants

Seed stocks	N/A
Novel plant genotypes	N/A
Authentication	N/A
